# THE FOUR C’S OF TEACHING OLDER LEARNERS: IMPLICATIONS FOR THE WORKPLACE AND CAREER WORK–LIFE EXTENSION EFFORTS

**DOI:** 10.1093/geroni/igad104.1032

**Published:** 2023-12-21

## Abstract

From Spring 2017 through Spring 2022, a sample of older (age 40 and above) undergraduate and graduate students who were also first-time online students (n = 86) from a very diverse student population at Northeastern Illinois University were analyzed on various motivational and performance indices regarding fully online course performance (e.g., student expectations, receptivity to on-line modules, time management skills). A comparative analysis of the two student samples yielded some interesting “universal” course design issues across education levels, as well as by cultural background and gender differences. Specific fully online design issues to be discussed as are how to create the clearest “face-to-face” representation of the classroom experience via a course learning management system. Effective design issues will be discussed in creating a supportive, fully online course environment for older online course students. General design issues to be discussed are how to create a well-structured “face-to-face” representation of the classroom experience for a diverse older student population with equally diverse learning needs/issues, a supportive fully online learning environment for “first-time” online course students, and tailored, adaptive learning environment to meet the dynamic needs of students (undergraduate, graduate) over the progress of an e-course. The presentation will present and discuss the four C’s in promoting a positive and enriched learning environment for diverse “non-traditional” adult learners faced with online learning in different contexts with implications for learning in the workplace for adaptive career work-life extension.

